# Supplementary material for: Predicting preterm birth using explainable machine learning in a prospective cohort of nulliparous and multiparous pregnant women
Source: PLoS One. 2023 Dec 27;18(12):e0293925. doi: 10.1371/journal.pone.0293925 (PMC10752564; doi:10.1371/journal.pone.0293925)
Supplement: S1 Table — (DOCX) [file pone.0293925.s001.docx]

**S1 Table: Features selection from the literature of potentials risk factors of preterm birth**

| Author | Total features | Features Used | Combination of Features |
| --- | --- | --- | --- |
| Diaz et al. ^1^ | 30 | **Sleep habits**: (Device Monday-Friday), Device SS, Interruption, etc  **Maternal:**  Age, BMI, obstetric history (primiparity and primigravity), weight at the beginning of pregnancy (kg), weight gain thought pregnancy (kg), multiple gestations (yes or not), toxic habits (tobacco or alcohol), Group B Streptococcus agalactiae carriage (yes or not)).  **Fetal variables**  sex, Nuchal translucency analysis in the first trimester (NT) (mm), estimated weight in the third trimester (g), weight at birth (g), folic acid administration (yes or not), Test O’Sullivan (normal or altered), hemoglobin concentration at the first trimester of pregnancy (g/dL), cervix dilatation (cm), premature rupture of membranes (PRM) (yes or not). | **Sleep habits**: (Device Monday-Friday), Device SS, Interruption, etc  **Maternal:**  Age, BMI, obstetric history (primiparity and primigravity), weight at the beginning of pregnancy (kg), weight gain thought pregnancy (kg), multiple gestation (yes or not), toxic habits (tobacco or alcohol), Group B Streptococcus agalactiae carriage (yes or not)).  **Fetal variables**  sex, Nuchal translucency analysis in the first trimester (NT) (mm), estimated weight in the third trimester (g), weight at birth (g), folic acid administration (yes or not), Test O’Sullivan (normal or altered), hemoglobin concentration at the first trimester of pregnancy (g/dL), cervix dilatation (cm), premature rupture of membranes (PRM) (yes or not).  Race, Marital status, Education (8th grade or less to doctorate), Number of previous terminations, Special supplemental nutrition program (WIC), Smoking before pregnancy, Height (inches), Weight (pounds), Parity  Pregnancy history, Pre-pregnancy diabetes  Gestational diabetes, Pre-pregnancy hypertension  Gestational hypertension, Hypertension eclampsia  Previous preterm births, Infertility treatment  Infertility drugs, Assisted reproductive technology (ART), Previous cesarean sections, Infections  Gonorrhea, Syphilis, Chlamydia, Hepatitis B, Hepatitis C  Cervical length, drinker (no, yes),  smoker (no, yes), diabetes mellitus (no, yes),  hypertensive disorder (no, yes); in vitro fertilization (no, yes), myomas & adenomyosis (no, yes), prior cone biopsy (no, yes), pelvic inflammatory disease history (no, yes), prior preterm birth (no, yes), prior placenta previa (no, yes).  **Demographic factors**  Age <16 yr, Age >35 yr  Race (black vs nonblack), Live along, <12 yr school  **Socioeconomic status**  Low family income (<$9600/yr), Poor home condition, Poor social environment, Work environment, Paying job during pregnancy  Medical history, Medical risk factors, Chronic pulmonary disease, Genital tract abnormalities or surgery, Pelvic inflammatory disease, Weight <100 lb before pregnancy, Obstetric history, Prior spontaneous preterm delivery (% yes), Prior term delivery (% no), Drug or alcohol abuse, Smoked cigarettes during pregnancy, Drank alcohol during pregnancy, Illicit drug use during pregnancy,  Complications of current gestation, Vaginal bleeding (first or second trimester), Any uterine contractions in past 2 wk, Symptoms suspicious for preterm labor, Acute pulmonary disease requiring treatment, Positive cultures, Urinary tract colonization or infection, Other infections  Group B Streptococcus colonization, Treatments or medications, Hospitalization (preterm contractions), Hospitalization (other indication)  Therapeutic restriction of activity, Antimicrobial therapy, Tocolytic therapy, Bronchodilator therapy, No prenatal vitamins, Physical examination: General, BMI <19.8  Physical examination: Digital, Cervix <1 cm long  Internal cervical os >1 cm, Cervix consistency soft (vs medium of firm), Bishop score (>4)  Patient identification, Woman age, Last menstrual period, Estimated delivery date, Gravida, Abortion, L Living, Educational level, Weight, Blood pressure, Hemoglobin, Antenatal care visits, Actual delivery date, Obstetric history, Previous caesarean section  Gestational age, Birth weight, Fetal heart rate, Multiple gestation, Normal delivery, Previous medical history, Low birth weight, Asphyxia Hypertension  Preeclampsia, Live birth, Still birth, Obesity, Anemia, TH (thyroid, Neonatal status  **Physical examination:**  Waist size, Fundal height, SBP, mmHg, DBP, mmHg, FHR, times/min, Weight, Edema  **Blood test:**  BG, Blood RH, serum albumin, g/L, ALT, U/L  alanine transaminase, U/L, Glu, mmol/L, total calcium, mmol/L, creatinine, umol/L, direct bilirubin, umol/L, total serum iron, umol/L globulins, g/L magnesium, mmol/L serum norganic phosphorus, mmol/L total biliary acid, umol/L total bilirubin, umol/L total cholesterol, mmol/L total protein, g/L triglycerides, mmol/L Urea, mol/L uric acid, umol/L basophil granulocytes, 10e9/L platelet count, 10e9/L eosinophil, granulocytes, 10e9/L hemoglobin, g/L intermediate cell, lymphocytes, 10e9/L mean cell hemoglobin, pg mean corpuscular hemoglobin concentration, g/L mean cell volume, fL monocytes, 10e9/L mean platelet volume, fL neutrophil granulocytes, 10e9/L mean platelet volume, % hematocrit, % plateletcrit, % platelet distribution width, % red blood cell distribution width-CV, red blood cell distribution width-CV, fL RBC, 10e12L WBC, 10e9/L  **Urine Test Strip:**  Urine pH, urine, specific gravity, urine bilirubin  Glycosuria, urine ketone bodies, Nitrituria, Blood Proteinuria, Bilirubinuria, Urine WBC  **Gynecological examination**  bacterial vaginosis, cleaning degree of vagina  vaginal yeast infection,  Neighborhood income quartile  Neighborhood education quartile, Neighborhood minority quartile, Neighborhood immigration quartile, Smoking status, Ex-smoker, Alcohol consumption, Drug (substance) use, First-trimester visit, Antenatal health care provider  Folic acid use, Intention to breastfeed, Pre-existing health conditions, Pre-existing mental health conditions, Number of previous abortions (including miscarriages), Conception type, Gravidity, Diabetes, Gestational weight gain during the first trimester, Pregnancy-associated plasma protein A, Free beta-subunit of human chorionic gonadotropin, Nuchal translucency  Dimeric inhibin A, Unconjugated estriol, Human chorionic gonadotropin, Alpha-fetoprotein, Diabetes during the second trimester, Hypertensive disorder, Infection(s), Medication exposure, Sex of fetus, Complications during pregnancy |
| Koivu et al.^2^ | 26 | Age (years), Race, Marital status, Education (8th grade or less to doctorate), Number of previous terminations, Special supplemental nutrition program (WIC), Smoking before pregnancy  BMI, Height (inches), Weight (pounds), Parity  Pregnancy history, Pre-pregnancy diabetes  Gestational diabetes, Pre-pregnancy hypertension  Gestational hypertension, Hypertension eclampsia  Previous preterm births, Infertility treatment  Infertility drugs, Assisted reproductive technology (ART), Previous cesarean sections, Infections  Gonorrhea, Syphilis, Chlamydia, Hepatitis B, Hepatitis C |  |
| Lee et al. ^3^ | 13 | age; BMI, Cervical length, Parity, drinker (no, yes),  smoker (no, yes), diabetes mellitus (no, yes),  hypertensive disorder (no, yes); in vitro fertilization (no, yes), myomas & adenomyosis (no, yes), prior cone biopsy (no, yes), pelvic inflammatory disease history (no, yes), prior preterm birth (no, yes), prior placenta previa (no, yes). |  |
| Mercer et al. ^4^ | 43 | **Demographic factors**  Age <16 yr , Age >35 yr  Race (black vs nonblack), Live along, <12 yr school  **Socioeconomic status**  Low family income (<$9600/yr), Poor home condition, Poor social environment, Work environment, Paying job during pregnancy  Medical history, Medical risk factors, Chronic pulmonary disease, Genital tract abnormalities or surgery, Pelvic inflammatory disease, Weight <100 lb before pregnancy, Obstetric history, Prior spontaneous preterm delivery (% yes), Prior term delivery (% no), Drug or alcohol abuse, Smoked cigarettes during pregnancy, Drank alcohol during pregnancy, Illicit drug use during pregnancy,  Complications of current gestation, Vaginal bleeding (first or second trimester), Any uterine contractions in past 2 wk, Symptoms suspicious for preterm labor, Acute pulmonary disease requiring treatment, Positive cultures, Urinary tract colonization or infection, Other infections  Group B Streptococcus colonization, Treatments or medications, Hospitalization (preterm contractions), Hospitalization (other indication)  Therapeutic restriction of activity, Antimicrobial therapy, Tocolytic therapy, Bronchodilator therapy, No prenatal vitamins, Physical examination: General, Body mass index <19.8  Physical examination: Digital, Cervix <1 cm long  Internal cervical os >1 cm, Cervix consistency soft (vs medium of firm), Bishop score (>4) |  |
| Raja et al. ^5^ | 35 | Patient identification, Woman age, Last menstrual period, Estimated delivery date, Gravida, Parity  Abortion, L Living, Educational level, Height, Weight, BMI, Blood pressure, Hemoglobin, Antenatal care visits, Actual delivery date, Obstetric history, Previous caesarean section  Gestational age, Birth weight, Gestational diabetes mellitus, Fetal heart rate, Multiple gestation, Normal delivery, Previous medical history, Low birth weight, Asphyxia Hypertension  Preeclampsia, Live birth, Still birth, Obesity, Anemia, TH (thyroid, Neonatal status |  |
| Sun et al. ^6^ | 60 | **Physical examination:**  Waist size, Fundal height, SBP, mmHg, DBP, mmHg  FHR, times/min, Weight, Edema  **Blood test:**  BG, Blood RH, serum albumin, g/L, ALT, U/L  alanine transaminase, U/L, Glu, mmol/L, total calcium, mmol/L, creatinine, umol/L, direct bilirubin, umol/L, total serum iron, umol/L globulins, g/L magnesium, mmol/L serum norganic phosphorus, mmol/L total biliary acid, umol/L total bilirubin, umol/L total cholesterol, mmol/L  total protein, g/L triglycerides, mmol/L Urea, mol/L uric acid, umol/L basophil granulocytes, 10e9/L platelet count, 10e9/L eosinophil, granulocytes, 10e9/L hemoglobin, g/L  intermediate cell, lymphocytes, 10e9/L mean cell hemoglobin, pg mean corpuscular hemoglobin concentration, g/L mean cell volume, fL monocytes, 10e9/L mean platelet volume, fL  neutrophil granulocytes, 10e9/L mean platelet volume, % hematocrit, % plateletcrit, %  platelet distribution width, % red blood cell distribution width-CV, red blood cell distribution width-CV, fL RBC, 10e12L WBC, 10e9/L  **Urine Test Strip:**  Urine pH, urine, specific gravity, urine bilirubin  Glycosuria, urine ketone bodies, Nitrituria, Blood  Proteinuria, Bilirubinuria, Urine WBC  **Gynecological examination**  bacterial vaginosis, cleaning degree of vagina  vaginal yeast infection, |  |
| Belaghi et al. ^7^ | Prediction of preterm birth in nulliparous women using logistic regression and machine  learning. | Age, Height, BMI, Neighborhood income quartile  Neighborhood education quartile, Neighborhood minority quartile, Neighborhood immigration quartile, Smoking status, Ex-smoker  Alcohol consumption, Drug (substance) use, First-trimester visit, Antenatal health care provider  Folic acid use, Intention to breastfeed, Pre-existing health conditions, Pre-existing mental health conditions, Number of previous abortions (including miscarriages), Conception type, Gravidity, Diabetes, Gestational weight gain during the first trimester, Pregnancy-associated plasma protein A, Free beta-subunit of human chorionic gonadotropin, Nuchal translucency  Dimeric inhibin A, Unconjugated estriol, Human chorionic gonadotropin, Alpha-fetoprotein, Diabetes during the second trimester, Hypertensive disorder, Infection(s), Medication exposure, Sex of fetus, Complications during pregnancy |  |

**REFERENCES :**

1. Díaz, E. *et al.* Machine learning as a tool to study the influence of chronodisruption in preterm births. *J. Ambient Intell. Humaniz. Comput. 2021 131* **13**, 381–392 (2021).

2. Koivu, A. & Sairanen, M. Predicting risk of stillbirth and preterm pregnancies with machine learning. *Health Inf. Sci. Syst.* **8**, 14 (2020).

3. Lee, K. S. & Ahn, K. H. Artificial Neural Network Analysis of Spontaneous Preterm Labor and Birth and Its Major Determinants. *J. Korean Med. Sci.* **34**, (2019).

4. Mercer, B. M. *et al.* The preterm prediction study: A clinical risk assessment system. *Am. J. Obstet. Gynecol.* **174**, 1885–1895 (1996).

5. Raja, R., Mukherjee, I. & Sarkar, B. K. A Machine Learning-Based Prediction Model for Preterm Birth in Rural India. *J. Healthc. Eng.* **2021**, (2021).

6. Sun, Q. *et al.* Machine Learning-Based Prediction Model of Preterm Birth Using Electronic Health Record. *J. Healthc. Eng.* **2022**, 1–12 (2022).

7. Belaghi, R. A., Beyene, J. & McDonald, S. D. Prediction of preterm birth in nulliparous women using logistic regression and machine learning. *PLOS ONE* **16**, e0252025 (2021).
