# Supplementary material for: Predicting preterm birth using explainable machine learning in a prospective cohort of nulliparous and multiparous pregnant women
Source: PLoS One. 2023 Dec 27;18(12):e0293925. doi: 10.1371/journal.pone.0293925 (PMC10752564; doi:10.1371/journal.pone.0293925)
Supplement: S2 Table — (DOCX) [file pone.0293925.s002.docx]

**S2 Table: Selected variables for prediction of preterm birth.**

| Outcome: Preterm Birth | <37 weeks of gestation |
| --- | --- |
| **Self-reported characteristics** |  |
| Planning status for this pregnancy | Binary (0= No, 1= Yes) |
| Employment | Binary (0= No, 1= Yes) |
| Education level | Binary (0= ≤ grade 12, 1= > grade 12) |
| Consanguinity | Binary (0= No, 1= Yes) |
| Passive smoking | Binary (0= No, 1= Yes) |
| Physical activity (PA) before pregnancy | Categories (0= Never, 1= 1-2 times/week, 2= 3-5 times/week, 3= daily) |
| Physical activity (PA) during pregnancy |  |
| Housing | Binary (0= Rent, 1= Owned) |
| Worry about upcoming childbirth | Binary (0= No, 1= Yes) |
| Previous infertility treatment | Binary (0= No, 1= Yes) |
| **Pregnancy and delivery characteristics** |  |
| Age | Continuous variable |
| Gravida | Count (Number of pregnancies including this one) |
| Parity | Count (Number of previous viable delivery) |
| Gestational age at delivery | Continuous variable |
| BMI at delivery | Continuous variable |
| Inter-pregnancy interval (days) | Continuous variable |
| Pre-existing hypertension | Binary (0= No, 1= Yes) |
| Pre-existing diabetes mellitus | Binary (0= No, 1= Yes) |
| History of previous preterm birth | Binary (0= No, 1= Yes) |
| History of previous cesarian delivery (CS) | Binary (0= No, 1= Yes) |
| History of previous pregnancy loss | Binary (0= No, 1= Yes) |
| Rh antibodies | Binary (0= Negative, 1= Positive) |
| Preeclampsia | Binary (0= No, 1= Yes) |
| Gestational diabetes mellitus | Binary (0= No, 1= Yes) |
| Fetal growth retardation | Binary (0= No, 1= Yes) |
| Antepartum haemorrhage | Binary (0= No, 1= Yes) |
| Polyhydramnios | Binary (0= No, 1= Yes) |
| Oligohydramnios | Binary (0= No, 1= Yes) |
| Infection of amniotic sac and membranes | Binary (0= No, 1= Yes) |
| Premature rupture of membranes | Binary (0= No, 1= Yes) |
| Placental disorders | Binary (0= No, 1= Yes) |
| Placenta praevia | Binary (0= No, 1= Yes) |
| Streptococcus B carrier | Binary (0= No, 1= Yes) |
| Genitourinary tract infection during pregnancy | Binary (0= No, 1= Yes) |
| Baby gender | Binary (0= Male, 1= Female) |
