# Supplementary material for: Predicting preterm birth using explainable machine learning in a prospective cohort of nulliparous and multiparous pregnant women
Source: PLoS One. 2023 Dec 27;18(12):e0293925. doi: 10.1371/journal.pone.0293925 (PMC10752564; doi:10.1371/journal.pone.0293925)
Supplement: S1 File — (DOCX) [file pone.0293925.s003.docx]

## **S1 File: Definition of SHAP and LIME algorithms.**

## **SHapley Additive exPlanations (SHAP) algorithm**

SHapley Additive exPlanations (SHAP) first introduced by Lundberg and Lee [40] and is based on

Shapley. The goal of SHAP is to explain the prediction of an instance x by computing the contribution

of each feature to the prediction. The SHAP method computes the Shapley values. In this case, the

feature values act as "players" in a coalition. Shapley values then tell us the fair distribution among the

features. Consider, a game, G consists of N players. Let C be the coalition of players and v(C) is the cost obtained from the coalition. Then for individual player $i$ from the cost function v, the Shapley value Ø can be obtained using:

$$Ø_{i}\left( v \right)=\frac{\sum_{\pi\epsilon C_{N}} (v(C\left( \pi,i \right)-(v\left( \frac{\pi,i}{i} \right))}{N!}$$

Where π represents the set of permutations, $C\left( \pi,i \right)$ is the set of players in the coalition. The higher the $Ø_{i}\left( v \right)$ the larger will be the payoff of the individual player.

Similarly, the game is analogous to the proposed PTB prediction model where each player is an input feature if the Shape value is higher for a specific feature, it means that the specific feature is contributing more to the diagnosis of GDM. The SHAP values obtained using $Ø_{i}\left( v \right)$ is a single numerical value that shows the impact of the ith feature on the model prediction.

Considering a model $f$ and set of input features $x$, the Shapley values estimation has three desirable properties ^8^ namely local accuracy, missingness, and consistency represented as:

$$f\left( x \right)=Ø_{0}+\sum_{i=1}^{N} Ø_{i}x_{i}$$

Where $Ø_{0}$ is the model output over the training dataset when $N$ is the number of interpretable inputs.

$\boldsymbol{f}_{\boldsymbol{x}}^{\boldsymbol{a}}\boldsymbol{(}$ $\boldsymbol{z}^{\boldsymbol{'}}\boldsymbol{)-}\boldsymbol{f}_{\boldsymbol{x}}^{\boldsymbol{a}}\boldsymbol{(}$ $\boldsymbol{z}^{\boldsymbol{'}}\boldsymbol{\backslash i)\geq}\boldsymbol{f}_{\boldsymbol{x}}^{\boldsymbol{b}}\boldsymbol{(}$ $\boldsymbol{z}^{\boldsymbol{'}}\boldsymbol{)-}\boldsymbol{f}_{\boldsymbol{x}}^{\boldsymbol{b}}\boldsymbol{(}$ $\boldsymbol{z}^{\boldsymbol{'}}\boldsymbol{\backslash i)}$

Where $f^{a}$ and $f^{b}$ are two models. Equation 3 shows that a feature was more important in $f^{a}$ then $f^{b}$. It shows that if a feature is important in more models then it should be important it should be also higher in the other model even if another feature is present i.e. it should be consistence. The two properties are provided using Shapely values. Missingness property represents that if $x_{i}=0$ then it will have no impact i.e. $Ø_{i}=0$.

**Local Interpretable Model-Agnostic Explanations (LIME) algorithm**

The interpretable explanations need to be understandable by humans even if the actual representation of the features is complex ^9^. LIME (Local Interpretable Model-Agnostic Explanations) explain a prediction by replacing the complex model with a locally interpretable surrogate model. For instance, in cat vs dog classification, let us classify that if the image contains a cat or not. Then actual the actual representation of an image by a model is a tensor of RGB color per pixel, however, the interpretation to humans is whether the image contains a cat or not. Mathematically, we can express the original representation of the image $x$ as $x \in\mathbb{R}^{d}$ while the interpretation to human can be represented as $x\in\{0,1\}$, for 1 means presence of cat while 0 means absence. To explain an instance $x$ using a model $g$ for $g\in G$ , where $G$ is explanation models such as decision trees, linear regression, etc. Then LIME based explanation can be obtained using Equation 6.

$$explnation\left( x \right)={argmin}_{g\in G}\mathcal{L}\left( f, g,\pi_{x} \right)+\Omega(g)$$

Since some models are more complex therefore the complexity term $\Omega(g)$ is used for the complexity of $g$, while $f_{x}$ is the original prediction model such as XGBoost. The locality around $x$ is measured by $\pi_{x}$. The loss $\mathcal{L}$ must be minimized to measure the error between the original model and the explanation.

LIME for tabular data follows the following simple steps:

1. Any instance $x_{interest}$ which is required to be explained to the human user is selected.
2. The dataset is perturbed and prediction by the black-box model is obtained.
3. The proximity of the new samples is calculated with the $x_{interest}$. For tabular data, the samples are taken from the training data mass center.
4. Interpretable and the weighted model are trained on the dataset.
5. Explanations are provided by the interpretable model.
