# Supplementary figures and images for: Predicting preterm birth using explainable machine learning in a prospective cohort of nulliparous and multiparous pregnant women
Source: PLoS One. 2023 Dec 27;18(12):e0293925. doi: 10.1371/journal.pone.0293925 (PMC10752564; doi:10.1371/journal.pone.0293925)

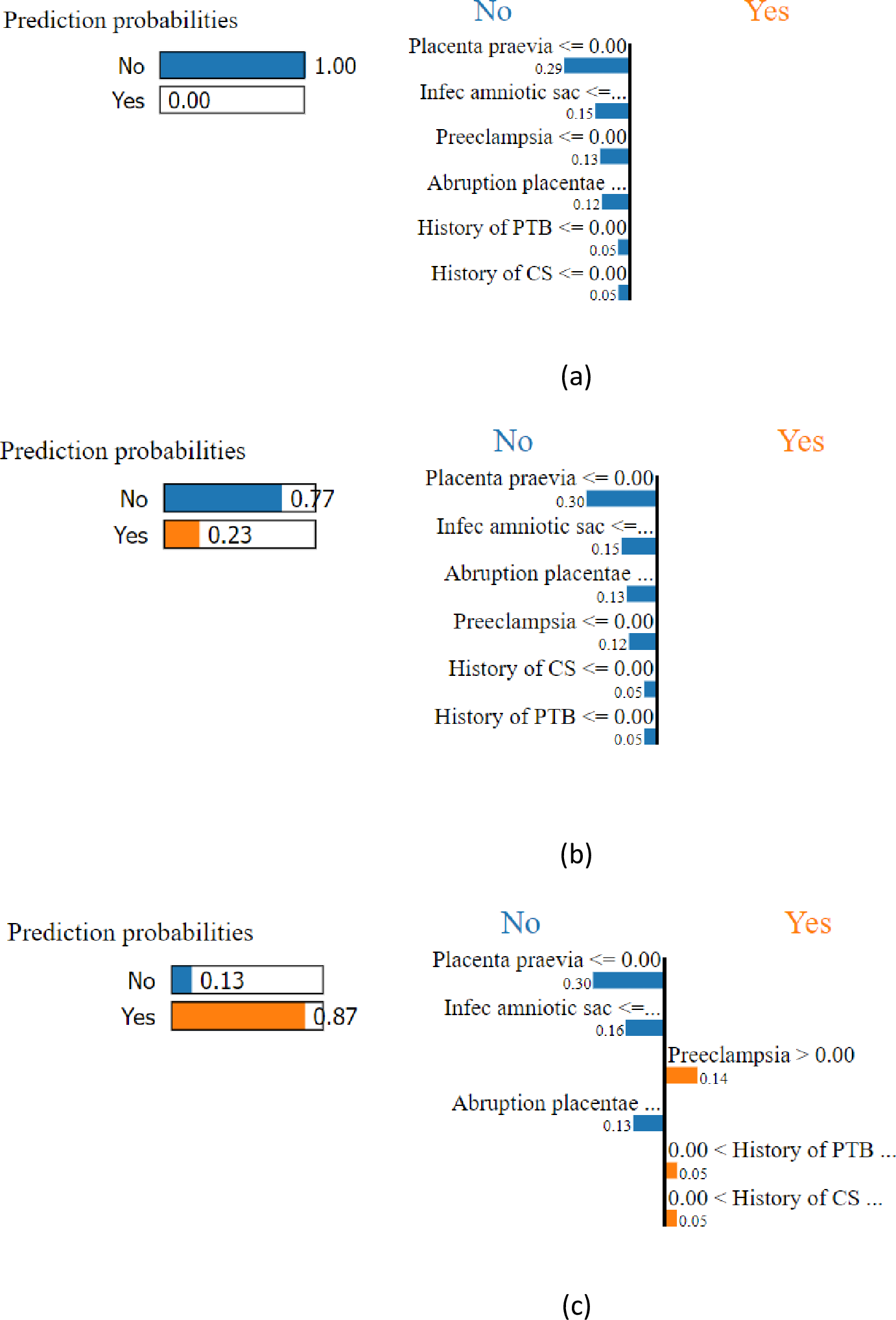

Supplement: S1 Fig — LIME prediction contains two parts: the left-side probability of the patient having PTB, and the right-side local explanation of the risk factors for a specific patient. Patient (a) is at a lower risk, patient (b) is at a median risk, and patient (c) is at a higher risk of PTB. (TIF) [file pone.0293925.s004.tif]

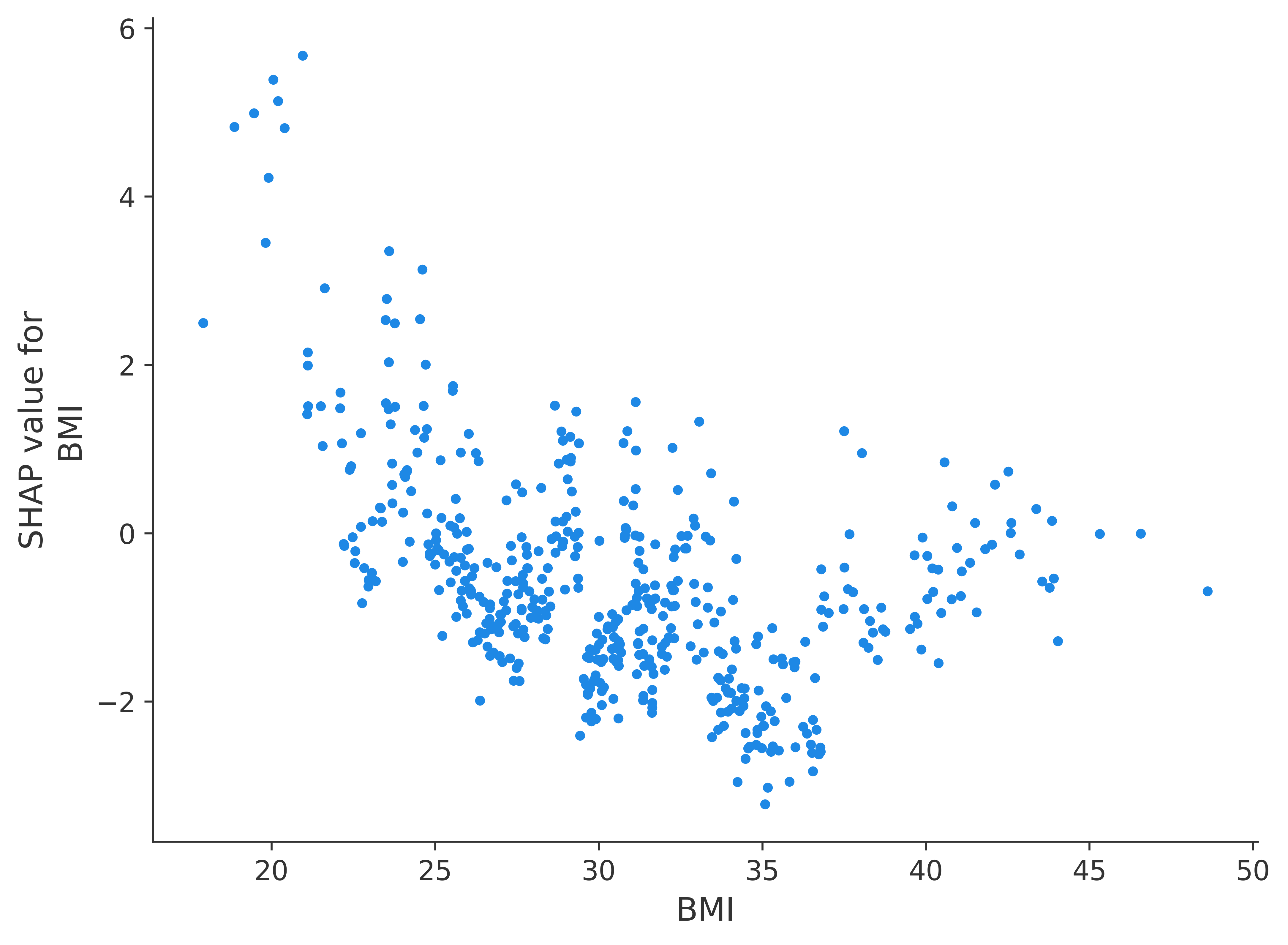

Supplement: S2 Fig — (TIF) [file pone.0293925.s005.tif]

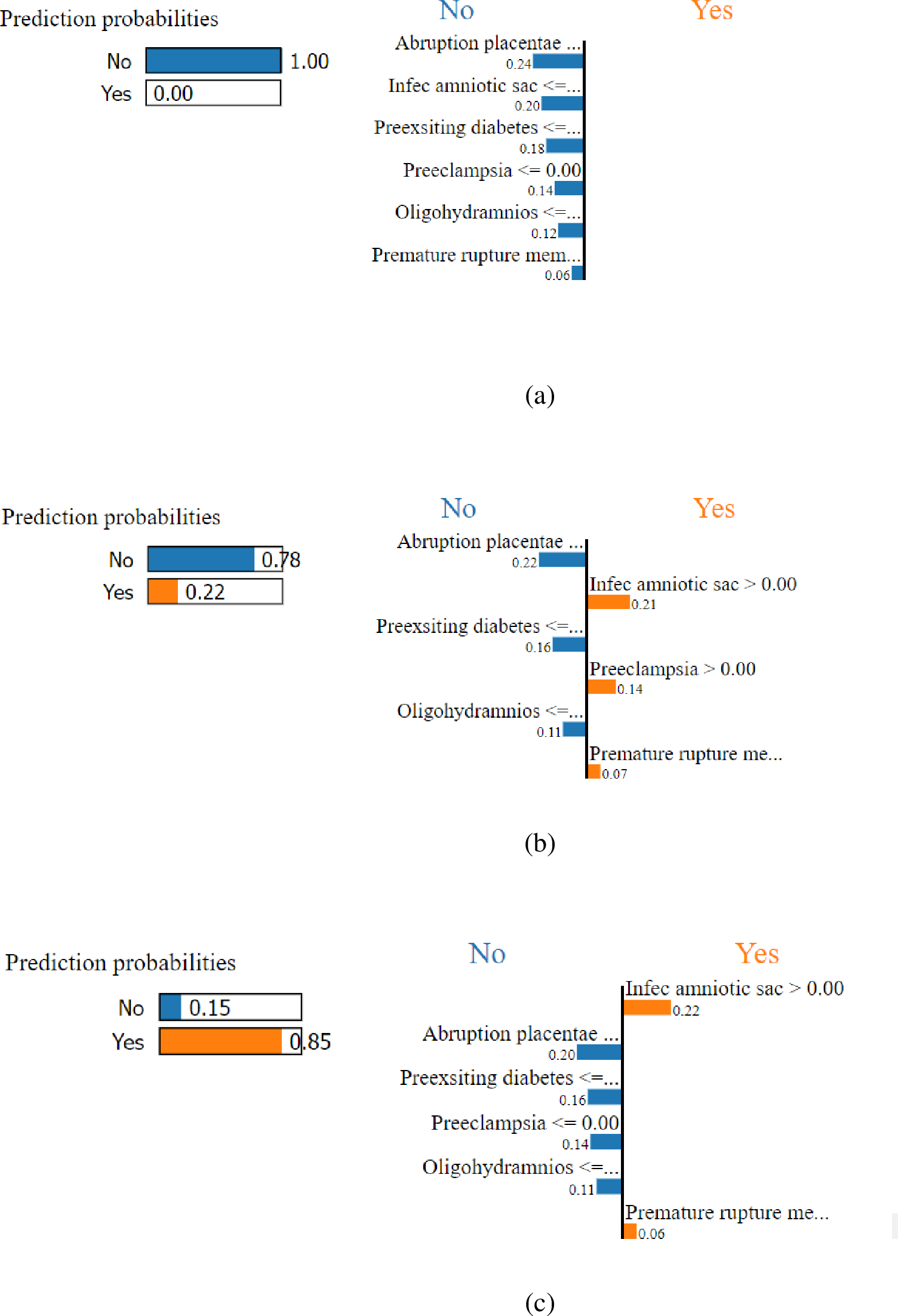

Supplement: S3 Fig — LIME prediction contains two parts: the left-side probability of the patient having PTB, and the right-side local explanation of the risk factors for a specific patient. Patient (a) is at a lower risk, patient (b) is at a median risk, and patient (c) is at a higher risk of PTB. (TIF) [file pone.0293925.s006.tif]
