# Supplementary material for: Predicting preterm birth using explainable machine learning in a prospective cohort of nulliparous and multiparous pregnant women
Source: PLoS One. 2023 Dec 27;18(12):e0293925. doi: 10.1371/journal.pone.0293925 (PMC10752564; doi:10.1371/journal.pone.0293925)
Supplement: Attachment — Submitted filename: Response to Reviewers.docx [file pone.0293925.s007.docx]

Subject: Response to Reviewers and Minor Revisions for Submission of Manuscript Titled "Predicting Preterm Birth using Explainable Machine Learning in a Prospective Cohort of Pregnant Women" to PLOS ONE

2 August 2023

Dear Editor

I hope this email finds you well. We would like to express our sincere gratitude to you and the esteemed reviewers for your thoughtful review of our manuscript titled "Predicting Preterm Birth using Explainable Machine Learning in a Prospective Cohort of Pregnant Women." We greatly appreciate the time and effort you and the reviewers have dedicated to providing valuable feedback to enhance the quality of our work.

We are pleased to inform you that we have addressed all the comments and suggestions from both the editor and the reviewers. Nearly all the recommendations have been carefully incorporated into the revised manuscript, resulting in significant improvements to the clarity, methodology, and presentation of our research.

A detailed point-by-point response to the comments, along with the revisions made, is provided in the appendix below.

Here is a summary of the major revisions made in response to the feedback:

Response to Editor's Comments:

1. The sentence about preterm birth etiology has been corrected to: "Preterm birth (PTB) is a multifactorial condition with complex causes" (Line 64).
2. "Low pre-pregnancy weight" has been replaced with "adolescent pregnancy" (Line 65).
3. Abbreviation "ML models" has been expanded to "Machine learning models" (Line 74).
4. All table abbreviations have been included below the respective tables.
5. The description of the analysis involving 3509 participants has been split into a new paragraph (Line 108-110).
6. The manuscript has been edited for grammar and language, as suggested.

Journal Requirements:

1. We have ensured that the manuscript adheres to PLOS ONE's style requirements, including proper file naming and formatting.
2. Regarding data availability, we have addressed the ethical restrictions by clarifying that while relevant data are available within the paper, the complete dataset cannot be made publicly available due to sensitive patient information. The complete dataset will be available upon justified request, following ethical approval from the Mutaba’ah Study ([mutabaah@uaeu.ac.ae](mailto:mutabaah@uaeu.ac.ae)).
3. Figure 1 has been modified to comply with PLOS ONE's copyright guidelines.
4. The reference list has been reviewed and verified for accuracy.

Reviewers' Comments:

1. The SHAP dependence plot for BMI vs. preterm birth for nulliparous women has been added as a supplementary file (S2 Fig) to clarify the association (Line 307-309).
2. The title has been updated to include "nulliparous and multiparous women" (Line 136).
3. Table 1 has been moved to the Methods section (Line 137).
4. The experimental setting has been improved and detailed in the Result Section (Lines 174-179).
5. Table 2 and Table 3 have been revised to present dichotomic responses more clearly.
6. Definitions and differences between "gravida" and "parity":

Gravida represents the total number of pregnancies a woman has had, while parity specifically refers to the number of pregnancies in which a woman has given birth to viable infants.

7. The description of the LIME methodology has been reduced, as recommended (Lines 169-171 deleted).
8. The characteristics of PTB and non-PTB women have been included in the results.
9. Clarifications have been made regarding the patient examples in Fig 3 and Fig 5 (a, b, c).

We believe that these revisions have significantly enhanced the manuscript's quality and addressed the concerns raised by the editor and reviewers. We are confident that our study's findings will make a valuable contribution to the field of predicting preterm birth using explainable machine learning.

Thank you once again for considering our manuscript for publication in PLOS ONE. We eagerly await your final decision and sincerely hope that the revised manuscript meets the standards of your esteemed journal. Should you require any further clarifications or information, please do not hesitate to contact us.

Best regards,

Ghenimi Nadirah, MD, PhD

Assistant Professor Family Medicine

(CMHS) P.O. Box No. 15551, Al Ain, UAE

T: 03-7137664 Email: [nghenimi@uaeu.ac.ae](mailto:nghenimi@uaeu.ac.ae)

**Appendix:**

The following sections of this cover letter provide a detailed point-by-point response to the comments raised by the editor and reviewers, along with a summary of the revisions made in the manuscript:

Response to Editor's Comments:

ACADEMIC EDITOR:

1) “Preterm birth (PTB) is a complex condition with multifactorial etiology “

Answer: We added reference and corrected the sentence for ` Preterm birth (PTB) is a multifactorial condition with complex causes’ (L64).

2) “e.g., advanced maternal age, low pre-pregnancy weight)” Both being very thin and being overweight are risk factors. Correct as: adolescent pregnancy, advanced maternal age….

Answer: We Added adolescent pregnancy instead of low-prepregnancy weight (L65).

3) Include the long version of the text in the first abbreviation. For example “ML models”

Answer: We replaced Machine learning models for ML models (L74).

4) Add all table abbreviations below the table.

Answer: We added all abbreviations definitions used under Table1, Table2 and Table3.

5) “This analysis included 3509” Split it into paragraphs.

Answer: Thank you for this suggestion. We split nulliparous and parous women into paragraphs in the methodology (L108-L110).

6) Please check the text and tables for grammar, seek professional grammar service if necessary!

Answer: Thank you and the manuscript was edited as suggested.

**Journal requirements:**

When submitting your revision, we need you to address these additional requirements.

1. Please ensure that your manuscript meets PLOS ONE's style requirements, including those for file naming. The PLOS ONE style templates can be found at

https://journals.plos.org/plosone/s/file?id=wjVg/PLOSOne_formatting_sample_main_body.pdf and

<https://journals.plos.org/plosone/s/file?id=ba62/PLOSOne_formatting_sample_title_authors_affiliations.pdf>

Answer: We have made the necessary revisions to ensure compliance with PLOS ONE's style requirements. We have thoroughly reviewed the manuscript's formatting and file naming, adhering to the journal's guidelines.

2. We note that you have indicated that data from this study are available upon request. PLOS only allows data to be available upon request if there are legal or ethical restrictions on sharing data publicly. For more information on unacceptable data access restrictions, please see http://journals.plos.org/plosone/s/data-availability#loc-unacceptable-data-access-restrictions.

In your revised cover letter, please address the following prompts:

a) If there are ethical or legal restrictions on sharing a de-identified data set, please explain them in detail (e.g., data contain potentially sensitive information, data are owned by a third-party organization, etc.) and who has imposed them (e.g., an ethics committee). Please also provide contact information for a data access committee, ethics committee, or other institutional body to which data requests may be sent.

b) If there are no restrictions, please upload the minimal anonymized data set necessary to replicate your study findings as either Supporting Information files or to a stable, public repository and provide us with the relevant URLs, DOIs, or accession numbers. For a list of acceptable repositories, please see http://journals.plos.org/plosone/s/data-availability#loc-recommended-repositories.

We will update your Data Availability statement on your behalf to reflect the information you provide.

Answer: Relevant data are available within the paper. Due to ethical restrictions, the complete dataset cannot be publicly available as the data contains potentially sensitive patient information. Data would be available upon justified request from the Mutaba’ah Study ([mutabaah@uaeu.ac.ae](mailto:mutabaah@uaeu.ac.ae)) after ethical approval.

3. We note that Figure 1 in your submission contains copyrighted images. All PLOS content is published under the Creative Commons Attribution License (CC BY 4.0), which means that the manuscript, images, and Supporting Information files will be freely available online, and any third party is permitted to access, download, copy, distribute, and use these materials in any way, even commercially, with proper attribution. For more information, see our copyright guidelines: http://journals.plos.org/plosone/s/licenses-and-copyright.

We require you to either (1) present written permission from the copyright holder to publish these figures specifically under the CC BY 4.0 license, or (2) remove the figures from your submission:

a. You may seek permission from the original copyright holder of Figure 1 to publish the content specifically under the CC BY 4.0 license.

b.If you are unable to obtain permission from the original copyright holder to publish these figures under the CC BY 4.0 license or if the copyright holder’s requirements are incompatible with the CC BY 4.0 license, please either i) remove the figure or ii) supply a replacement figure that complies with the CC BY 4.0 license.

Answer: Figure 1 was changed as per suggestion.

4. Please review your reference list to ensure that it is complete and correct. If you have cited papers that have been retracted, please include the rationale for doing so in the manuscript text, or remove these references and replace them with relevant current references. Any changes to the reference list should be mentioned in the rebuttal letter that accompanies your revised manuscript. If you need to cite a retracted article, indicate the article’s retracted status in the References list and also include a citation and full reference for the retraction notice.

Answer : Reference listing was verified as complete and correct.

**Reviewers' comments:**

Reviewer's Responses to Questions

Comments to the Author

5. Review Comments to the Author

Please use the space provided to explain your answers to the questions above. You may also include additional comments for the author, including concerns about dual publication, research ethics, or publication ethics. (Please upload your review as an attachment if it exceeds 20,000 characters)

Reviewer #1: I am really grateful for reviewing this manuscript. In my opinion, this manuscript can be published once some revision is done successfully. This study used 3509 pregnant women, six machine learning models and achieved the area under the curve of 72.3%-73.5% (boosting) for the prediction of preterm birth. This study employed SHAP (SHapley Additive exPlanations) as an explainable artificial intelligence approach. I would argue that this is a great achievement. However, it can be noted that the direction of association between body mass index and preterm birth is not very clear in the SHAP summary plot for nulliparous pregnant women. I would like to suggest the authors to derive the SHAP dependence plot for body mass index vs. preterm birth for nulliparous women, give that this would aid in clarifying the issue.

Answer: Thank you for your suggestion. The plot depicting the SHAP dependence plot for BMI was submitted as a supplementary file (S2 Fig). The figures displaying the low BMIs with higher SHAP values, clarify the differences between patient (a) and patient (c) in Fig 5 (L307-L309).

Reviewer #2: The manuscript describes the use of explainable machine learning to predict preterm birth through medical records and self-administered questionnaires. The authors separate nulliparous from multiparous. This must be declared in the title, for example, by adding ... "in nulliparous and multiparous women."

Answer: Thank you for this invaluable suggestion. We edited the title to include nulliparous, “Predicting preterm birth using explainable machine learning in a prospective cohort of nulliparous and multiparous women.”

The manuscript is easy to read.

Other comments:

Table 1, described in Introduction, could be moved to the Result Section since it is part of the study.

Answer: Table 1 was moved into the Methods section as it is not described in Results (L137).

The experimental setting provides little information due to everything explained before. Therefore, it may be better to improve this section and move it to Result Section.

Answer: The experimental setting was modified to include a detailed description (L174-L179).

The tables are too big; in dichotomic responses, put only one.

Answer: We agree with the reviewer. Table 2 and Table 3 were edited and the “No” was deleted. As the totals vary for the different variables, we added percentages in brackets for the “Yes”.

Which is the difference between Gravida and Parity?

Answer: Definitions and differences between "gravida" and "parity" in the context of obstetrics:

Gravida: Gravida refers to the number of times a woman has been pregnant, regardless of the outcome of the pregnancy. It includes both ongoing pregnancies and pregnancies that have ended in any outcome (live births, stillbirths, miscarriages, ectopic pregnancies, etc.). Gravida is a term used to indicate the total number of pregnancies a woman has experienced.

Reference: Cunningham, F. G., Leveno, K. J., Bloom, S. L., Spong, C. Y., Dashe, J. S., & Hoffman, B. L. (2018). Williams obstetrics. McGraw-Hill Education.

Parity: Parity refers to the number of pregnancies in which the foetus or foetuses have reached the stage of viability (at least 20 weeks of gestation) and have been born alive, regardless of whether the child is currently alive or deceased. It does not include ongoing pregnancies, stillbirths, or pregnancies that ended before 20 weeks. Parity is a term used to indicate the number of times a woman has given birth to a viable infant.

Reference: American College of Obstetricians and Gynecologists. (2018). ACOG Committee Opinion No. 736: Optimizing Postpartum Care. Obstetrics and Gynecology, 131(5), e140-e150.

In summary, gravida represents the total number of pregnancies a woman has had, while parity specifically refers to the number of pregnancies in which a woman has given birth to viable infants.

These are discrete quantitative variables, so the median and range are recommended.

Answer: Thank you for this suggestion and the authors agree that the variables, “gravida and parity” are discrete variables. The median and range was added to Table 2 (L210).

The authors describe LIME approximation, but all the results are in the supplementary figures and tables. Therefore, it is recommended to reduce LIME's description of methodology since the authors do not give the importance expected in the manuscript.

Answer: The authors agree and the LIME description in the methodology was edited as required (L169-L171 deleted).

In line 185, it is described as a different PTB with no PTB on maternal age, gravida, ... but it does not specify which characteristics are present in PTB. In line 193, it is the same question.

Answer: We added the characteristics of PTB women and no PTB women in the results.

The more significant characteristics for women who had experienced PTB is maternal age, gravida and, level of education passive smoking, infertility treatment, pre-existing hypertension and pre-existent Diabetes. `

In line 209, was BMI a qualitative or quantitative variable?

Answer : The BMI is a quantitative variable.

In line 211, it is unclear if the author put a patient as an example, as in (b) and (c).

Answer : Yes, we did use a patient in Fig 3 and Fig 5 a, b and c. These are 3 different patients described in the examples. We addressed this suggestion and edited this in the manuscript. The authors hope that this is now clearer to the reviewer.
