# Supplementary material for: Predicting preterm birth using explainable machine learning in a prospective cohort of nulliparous and multiparous pregnant women
Source: PLoS One. 2023 Dec 27;18(12):e0293925. doi: 10.1371/journal.pone.0293925 (PMC10752564; doi:10.1371/journal.pone.0293925)
Supplement: Attachment — Submitted filename: rev.docx [file pone.0293925.s008.docx]

1) “infection of the amniotic” It's not an accurate definition. Correct as “intra-amniotic infection” or “infection of the amniotic fluid” or “chorioamnionitis”.
2) Key words should not contain abbreviations.
3) “models lack proper interpretations for clinicians.” Correct as: “models lack proper interpretation for clinicians.”
4) “Preterm birth (PTB) complications is” Correct as: “Preterm birth (PTB) complications are”
5) “Hence, to be useful,“ Correct as: “Hence, for them to be useful”
6) Correct as: “with PTB were significantly different **from”**
7) Correct as: “infection **of the** amniotic sac”
8) Correct as: “and **a** low level of education. ”
9) Correct as: “and **a** history of previous PTB are”
10) Correct as: “conditions **such as** preexisting diabetes”
11) Correct as: “infection **of the** amniotic sac,”
12) Correct as: “patient (b) **is at median risk”**
13) Correct as: “risk of 0.22 **for** PTB”
14) Correct as: “factors in parous women were **having a** previous PTB”
15) Correct as: “maternal age, infection **of** the amniotic fluid,”
