# Supplementary material for: Predicting preterm birth using explainable machine learning in a prospective cohort of nulliparous and multiparous pregnant women
Source: PLoS One. 2023 Dec 27;18(12):e0293925. doi: 10.1371/journal.pone.0293925 (PMC10752564; doi:10.1371/journal.pone.0293925)
Supplement: Attachment — Submitted filename: Response to Reviewers.docx [file pone.0293925.s009.docx]

Subject: Response to Editor for Submission of Manuscript Titled

"Predicting Preterm Birth using Explainable Machine Learning in a Prospective Cohort of Pregnant Women" to PLOS ONE

15 October 2023

Dear Editor,

Thank you for the comprehensive feedback on our manuscript submitted to PLOS ONE. We appreciate the time and effort invested by the reviewers and the editorial team to evaluate our work. I acknowledge all the comments and have made the necessary revisions to address them. In the initial review process, minor revisions were requested, and we are pleased to report that we have successfully incorporated these changes.

In response to your feedback, we've engaged professional language and grammar editors to meticulously correct and enhance the quality, punctuation, and spelling in our manuscript. We've attached a certificate from this editing service for your reference.

Additionally, while revising our submission, we followed PLOS ONE's guidelines and uploaded our figure files to the Preflight Analysis and Conversion Engine (PACE) digital diagnostic tool.

The revised manuscript (with tracked changes), the clean manuscript, and this response letter have been uploaded to the submission portal as instructed.

With these revisions, we believe the manuscript now aligns with PLOS ONE's high standards. We appreciate the opportunity to improve our work and are eager for your feedback on the revised version.

Best regards,

Dr Ghenimi Nadirah, MD, PhD

Assistant Professor Family Medicine

(CMHS) P.O. Box No. 15551, Al Ain, UAE

T: 03-7137664 Email: [nghenimi@uaeu.ac.ae](mailto:nghenimi@uaeu.ac.ae)

Appendix :

Below is a point-by-point response to the comments:

Editor's Comments and Author's Responses:

1) Comment: “Infection of the amniotic” It's not an accurate definition. Correct as “intra-amniotic infection” or “infection of the amniotic fluid” or “chorioamnionitis”.

Response: We have corrected this phrase to "infection of the amniotic sac" throughout the manuscript. We used ICD10 code for a selection of the features from the Electronic medical records and the ICD10 code for intraamniotic infection is:

` 2024 ICD-10-CM Diagnosis Code O41.1090: `Infection of amniotic sac and membranes, unspecified, unspecified trimester, not applicable or unspecified`

2) Comment: Key words should not contain abbreviations.

Response: We have removed all abbreviations from the keywords. L 60 : Keywords: Preterm birth prediction, Machine Learning model, risk assessment, SHapley Additive exPlanations, Local interpretable model-agnostic explanations.

3) Comment: “models lack proper interpretations for clinicians.” Correct as: “models lack proper interpretation for clinicians.”

Response: We have made the suggested correction: L 28 the sentence was re-edited with ` While machine learning (ML) algorithms have shown promise in PTB prediction, the lack of interpretability in existing models hinders their clinical utility

4) Comment: “Preterm birth (PTB) complications is” Correct as: “Preterm birth (PTB) complications are”

Response: We have corrected this phrase as suggested.

5) Comment: “Hence, to be useful,“ Correct as: “Hence, for them to be useful”

Response: This phrase has been revised accordingly.

6) Comment: Correct as: “with PTB were significantly different from”

Response: The necessary changes have been made.

7-8) Comment: Multiple corrections suggesting usage of “infection of the amniotic sac” and "and a low level of education."

Response: These corrections have been applied in the manuscript as suggested.

9-15) Comment: Several phrasing corrections were provided.

Response: All the recommended phrasing corrections have been applied throughout the manuscript.
